# Glutamatergic axon-derived BDNF controls GABAergic synaptic differentiation in the cerebellum

**DOI:** 10.1038/srep20201

**Published:** 2016-02-01

**Authors:** Albert I. Chen, Keling Zang, Eliezer Masliah, Louis F. Reichardt

**Affiliations:** 1 University of California, San Francisco, Department of Physiology and Neuroscience Program, San Francisco, CA 94158, USA; 2 Nanyang Technological University, School of Biological Sciences and Warwick-NTU Neuroscience Programme, Singapore 138673; 3 University of Warwick, School of Life Sciences, Coventry CV4 7AL, UK; 4 Institute of Molecular and Cell Biology, Singapore 138673; 5 University of California, San Diego, Department of Neurosciences, San Diego, CA 92093, USA

## Abstract

To study mechanisms that regulate the construction of inhibitory circuits, we examined the role of brain-derived neurotrophic factor (BDNF) in the assembly of GABAergic inhibitory synapses in the mouse cerebellar cortex. We show that within the cerebellum, BDNF-expressing cells are restricted to the internal granular layer (IGL), but that the BDNF protein is present within mossy fibers which originate from cells located outside of the cerebellum. In contrast to deletion of *TrkB*, the cognate receptor for BDNF, deletion of *Bdnf* from cerebellar cell bodies alone did not perturb the localization of pre- or postsynaptic constituents at the GABAergic synapses formed by Golgi cell axons on granule cell dendrites within the IGL. Instead, we found that BDNF derived from excitatory mossy fiber endings controls their differentiation. Our findings thus indicate that cerebellar BDNF is derived primarily from excitatory neurons—precerebellar nuclei/spinal cord neurons that give rise to mossy fibers—and promotes GABAergic synapse formation as a result of release from axons. Thus, within the cerebellum the preferential localization of BDNF to axons enhances the specificity through which BDNF promotes GABAergic synaptic differentiation.

Neurotrophic factor signaling is essential for the development of the nervous system and the absence of neurotrophins or their receptors have been linked to developmental and behavioral disorders including depression, bipolar disorder, addiction, anxiety, obesity and many neurodegenerative diseases^1^. In particular, brain-derived neurotrophic factor (BDNF), the BDNF prohormone (proBDNF), and their receptors, TrkB and p75NTR, have been shown to regulate synapse formation and circuit function as molecular signals that also integrate synaptic activity^2^,^3^,^4^,^5^,^6^,^7^,^8^. TrkB and its ligands regulate many aspects of cerebellar development, including granule cell survival and migration, climbing fiber innervation, inhibitory synapse formation/maintenance and synaptic plasticity^9^,^10^,^11^,^12^,^13^,^14^. BDNF promotes migration of granule cells from the external to internal granular layer^15^. BDNF potentiates Purkinje cell responses to GABA^10^,^16^ and controls maturation of granule cell synapses through promoting NMDA receptor subunit switching^17^. Thus, BDNF and TrkB play an important role in the organization of the cytoarchitecture as well as connectivity within the cerebellar cortex and are critical determinants of cerebellar function.

Although TrkB can be activated by NT4 and, in some instances, NT3, BDNF is the most important ligand for TrkB within the brain^7^. BDNF transcription, translation, processing, and secretion are controlled, in part, by synaptic activity^4^,^5^,^18^,^19^,^20^,^21^; the surface expression of TrkB and signaling after BDNF binding are controlled through membrane trafficking processes that are also controlled, in part, via synaptic activity^22^,^23^. Both BDNF release and cerebellar development are impaired in mice lacking CAPS2, a MUNC13 homologue that promotes activity-dependent BDNF release^13^,^14^,^24^, suggesting that BDNF is an important effector in activity-stimulated cerebellar development and circuit maturation. Within the cerebellum, BDNF and TrkB are expressed at high levels postnatally, including the major period of synaptogenesis^12^,^25^. BDNF is expressed at high levels in the internal granular layer in the cerebellar cortex and in the deep cerebellar nuclei, while TrkB is expressed in Purkinje and granule cells, interneurons and glia^26^,^27^. *In vivo* and *in vitro* studies show that exogenous BDNF promotes inhibitory synaptogenesis, whereas inhibition of BDNF binding results in a decrease in inhibitory synapses in the cerebellum and hippocampus^28^,^29^. We previously showed that TrkB acts within GABAergic interneurons and their targets to regulate the formation and maintenance of inhibitory synapses and localization of proteins and organelles associated with these synapses^9^,^12^, however, the sources of BDNF involved in controlling GABAergic synapses within the cerebellar cortex were not determined in these studies. Moreover, the subcellular localization of BDNF in cell-types that express BDNF and whether BDNF exerts its activities through axons and/or dendrites remain controversial^14^,^18^,^19^,^30^,^31^.

## Results

### *Bdnf* expression is restricted to the internal granular layer and the protein is localized on granule cell axons and mossy fiber endings

**Figure 1:**
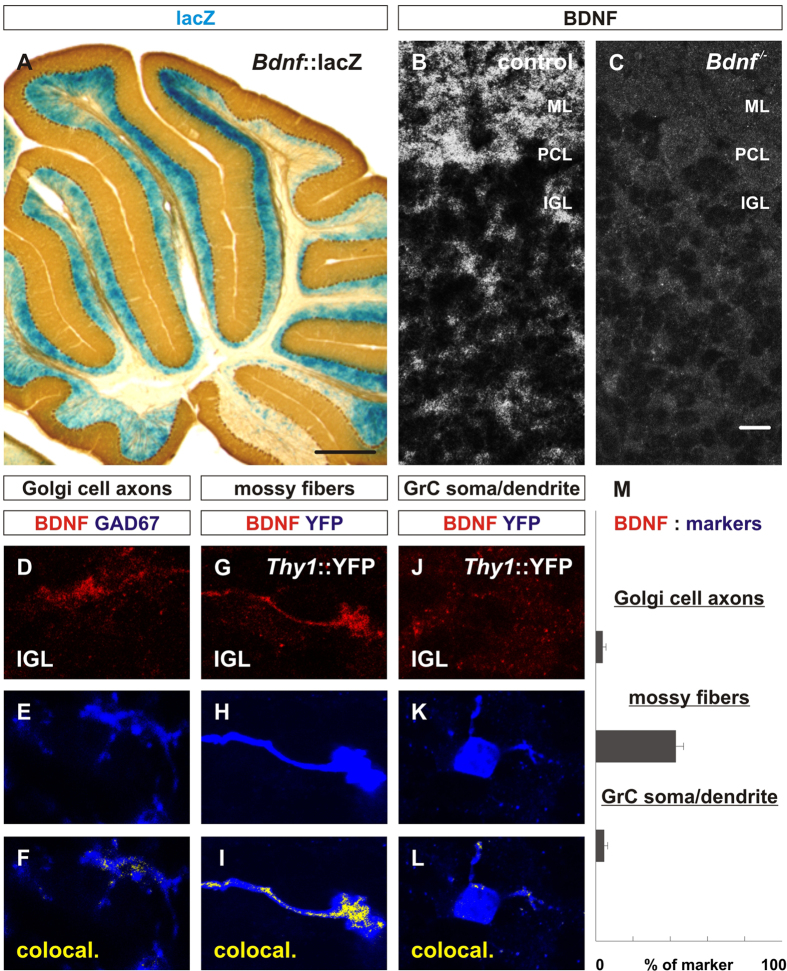
*Bdnf* is expressed by cells in the internal granular layer and the protein is localized throughout the cerebellar cortex. (**A**) The expression of BDNF is assessed by β-galactosidase reactivity in sagittal cerebellar sections obtained from three P30 *Bdnf*^*lacZ*^ mice^32^. LacZ expression is only detected in the internal granular layer (IGL) in sagittal cerebellar sections from *Bdnf*^*lacZ*^ mice. (**B,C**) Using a monoclonal mouse antibody (Mab#9) against mature BDNF, we examined the localization of BDNF protein in three P14 *Bdnf*^*−/−*^ mice compared to three control mice. (**B**) After antigen retrieval with sodium citrate, the BDNF protein is observed to be localized in the molecular layer, Purkinje cell layer and internal granular layer. (**C**) BDNF is lost in all layers of the cerebellar cortex in mice lacking *Bdnf*. (**D–F**) BDNF (red, E) is compared with GAD67 (blue, F) which labels Golgi cell terminals and does not appear to colocalize with Golgi cell axons (yellow, F). (**G–I**) When BDNF (red, G) is compared with vGluT1^+^ mossy fiber endings (blue, H), BDNF is extensively colocalized with mossy fiber endings (yellow, I). (**J–L**) BDNF (red, J) is compared with YFP (blue, K) which labels a subset of granule cells in *Thy1*::YFP mice. BDNF does not appear to colocalize with granule cell soma or dendrites (yellow, L). (**M**) Quantification of the area of BDNF on cell-type-specific markers indicate 4.33 ± 0.75% of GAD67^+^ Golgi cell axons contain BDNF, 43.08 ± 3.9% of vGluT1^+^ mossy fibers contain BDNF, 4.58 ± 0.84% of YFP^+^ granule cell soma/dendrites contain BDNF. Cerebella from four P21 wildtype mice and four P21 *Thy1*::YFP mice were used for analysis in D-M. Scale bar = 500 μm (A), 20 μm (B-P). *From herein, all statistics were done using Mann-Whitney U Test and errors represented as standard error of the mean.

To begin to assess the involvement of BDNF signaling in regulating cerebellar GABAergic synapses, we first examined the expression of *Bdnf* mRNA and BDNF protein. Consistent with studies by others, we observed prominent expression of *Bdnf*, detected with a lacZ knock-in, in the internal granular layer of the cerebellum^15^,^32^ (Fig. 1A). LacZ expression was not detected in the molecular layer, Purkinje cell layer or white matter layer (Fig. 1A). We used a previously described mouse monoclonal anti-BDNF antibody (Mab#9) directed against the mature domain of BDNF^31^ to show that the BDNF protein is much more widely distributed with localization in the molecular layer, Purkinje cell layer and internal granular layer of the cerebellar cortex (Fig. 1B–C), consistent with recent observations by others^14^. To confirm the specificity of the BDNF antibody, we used conventional *Bdnf* knockout mice as a negative control. As expected, BDNF protein appeared to be absent in the cerebella of mice lacking *Bdnf* (Fig. 1C).

To analyze the expression of BDNF protein in more detail, we compared the expression of BDNF with that of cell-type specific molecular markers using immunohistochemistry. We observed that within the internal granular layer, little or no BDNF localized to Golgi cell axons (Fig. 1D–F). In contrast, substantial BDNF was found on vGluT1^+^ mossy fiber terminals (Fig. G–I). Additionally, relatively little BDNF, but detectable amount, was found in the soma or dendrites of granule cells (Fig. 1J–L). Quantification of the area coverage of BDNF on specific cell compartments indicate that BDNF protein overlapped with less than 5% of the area of Golgi cell axons or granule cell dendrites/soma. In contrast, BDNF protein overlapped with ~43% of the area occupied by vGluT1^+^ mossy fiber terminals in the internal granular layer (Fig. 1M). Considering the 15–25 μm thickness of the sections used for these studies, the low overlap of BDNF with Golgi cell axons or granule cell dendrites/soma may reflect presence of BDNF protein outside of these structures.

**Figure 2:**
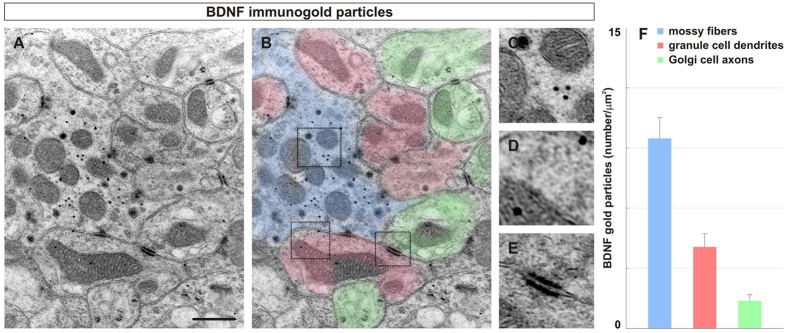
Immunogold particles of BDNF in the internal granular layer are primarily localized on mossy fiber endings. (**A–E**) We analyzed individual glomeruli from 20 electron micrographs obtained from four P60 wildtype mice (magnification 11,500X, single 2 μm sections) after immunogold labeling with BDNF Mab#9 antibody. (**A–B**) Individual components of a typical glomerulus (**A**) are color coded (B, mossy fibers = blue, granule cell dendrites = red, Golgi cell endings = green). (**C–E**) Regions correspond to dotted boxes. Most aggregates of gold grains are found in mossy fiber terminals (blue, B, C) while some gold grains are found in granule cell dendrites (red, B, D). Little gold clusters were found on Golgi cell axons (green, B, E). (**F**) Quantification of the number of gold particles per μm^2^ indicates 2.3X more gold particles were found in mossy fibers than in granule cell dendrites and 7.1X more gold particles than Golgi cell axons (MF = 9.41 ± 1.1/μm^2^ , granule cell = 4.08 ± 0.73/μm^2^, Golgi cell axons = 1.32 ± 0.31/μm^2^). Scale bar = 2 μm.

In order to confirm the localization of BDNF protein, electron micrographs of ultrathin sections of the internal granular layer were examined after labeling with an antibody against BDNF and visualization using secondary antibody-coated gold particles. The cerebellar glomeruli and major components of the glomeruli were identified based on their typical morphological characteristics previously described^9^,^33^,^34^. We found the highest density of BDNF within mossy fiber terminals (9.41 ± 1.1 particles/μm^2^) with a substantially lower density within Golgi cell axons (1.32 ± 0.31 particles/μm^2^) (Fig. 2A–F). Only a low density (4.08 ± 0.73 particles/μm^2^) of gold particles was present within granule cell dendrites (Fig. 2B,F). These results are consistent with the results shown in Fig. 1. Together, the data indicate that BDNF is primarily localized within mossy fiber terminals with much lower presence within Golgi cell axons and granule cell dendrites. Since both granule and Golgi cells express TrkB^12^, this could also be a result of receptor binding and endocytosis. Our data also show that even though granule cells express the *Bdnf* gene, BDNF expressed within granule cells is mostly directed to their axons, not their dendrites (see also ref.^14^). This is consistent with a preferential localization of BDNF to axons, but not dendrites of excitatory hippocampal and cortical neurons^6^,^30^,^31^. Finally, the most important source of BDNF protein in the IGL appears to be the axons and terminals of mossy fibers.

### Consequences of loss of BDNF on the localization of cerebellar GABAergic synaptic proteins

**Figure 3:**
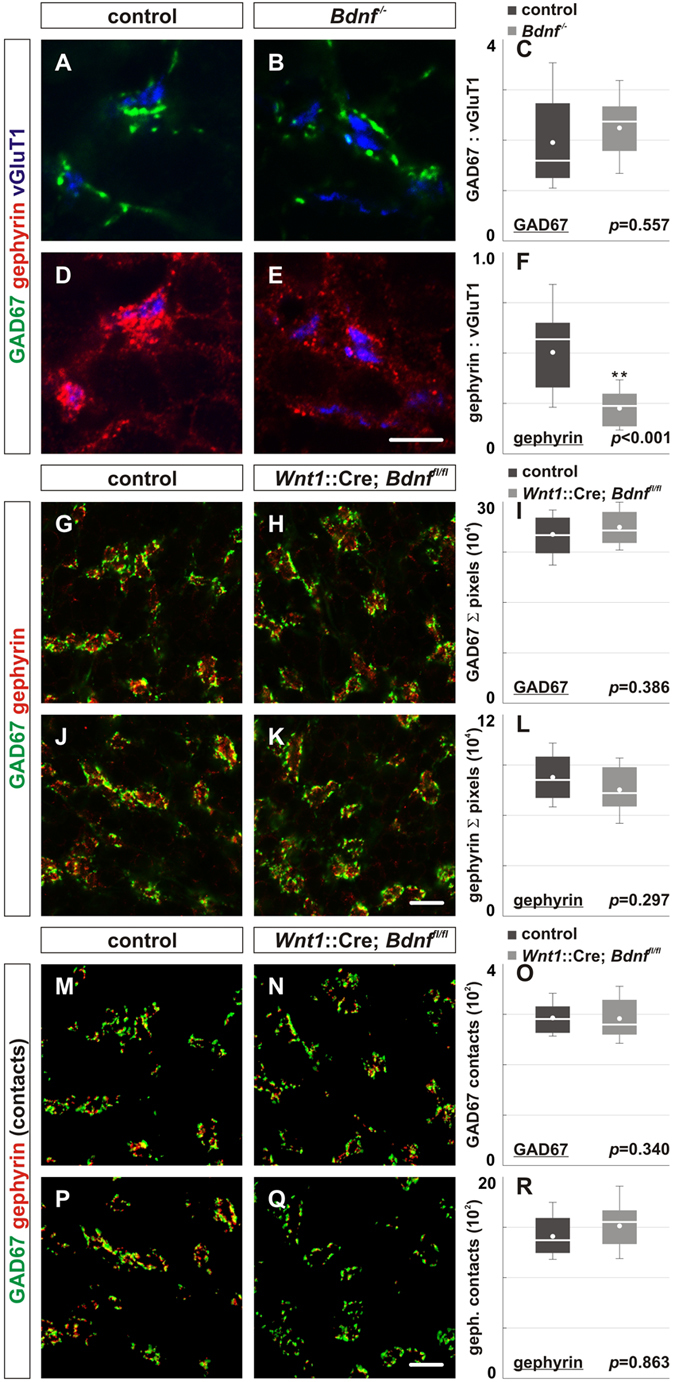
Consequences of complete versus specific deletion of *Bdnf* on the expression of GABAergic synaptic proteins and the number of contacts between these proteins in the internal granular layer. (**A–F**) We analyzed the localization of pre- and postsynaptic GABAergic synaptic proteins in P14 conventional *Bdnf*^*−/−*^ mice. (**A,B**) The expression of GAD67 (green) surrounding vGluT1^+^ mossy fibers (blue) is similar in *Bdnf*^*−/−*^ mice (**B**) compared to control (**A**). (**D,E**) However, the expression of gephyrin (red) is significantly reduced in *Bdnf*^*−/−*^ mice (**E**) compared to control (**D**). (**C,F**) Quantification of the area ratios of GAD67:vGluT1 and gephyrin:vGluT1 expression in *Bdnf*^*−/−*^ mice (GAD67: 2.88 ± 0.79; gephyrin: 1.07 ± 0.37) compared to control (GAD67: 2.95 ± 0.48, *p* = 0.557; gephyrin: 3.4 ± 0.39, *p* < 0.001). (**G–L**) Deletion of *Bdnf* in the cerebellum did not influence GAD67 (green) or gephyrin (red) localization in the IGL of P21 *Wnt1*::Cre; *Bdnf*^*fl*/*fl*^ mice (**G,J**) compared to control (**A,D**). (**I,L**) Quantification of the area of the coverage of GAD67 (**I**) and gephyrin expression (**L**) in control (GAD67: 25.1 ± 0.9 × 10^4^; gephyrin: 8.2 ± 0.5 × 10^4^) and *Wnt1*::Cre; *Bdnf*^*fl/fl*^ mice (GAD67: 26.2 ± 0.8, *p* = 0.386; gephyrin: 7.4 ± 0.4 × 10^4^, *p* = 0.297). (**M–R**) Similarly, loss of BDNF in the cerebellum did not influence the number of gephyrin contacts found on GAD67 puncta in the IGL of P21 *Wnt1*::Cre; *Bdnf*^*fl/fl*^ mice (**N,Q**) or the number of GAD67 contacts found on gephyrin puncta compared to control (**M,P**). (**O,R**) Quantification of the number of GAD67 contacts (# of gephyrin puncta on one GAD67 puncta) and the number of gephyrin contacts (# of GAD67 puncta on one gephyrin puncta) in control (GAD67 contacts: 2.79 ± 0.1 × 10^2^; gephyrin contacts: 14.1 ± 0.7 × 10^2^) and *Wnt1*::Cre; *Bdnf*^*fl/fl*^ mice (GAD67 contacts: 2.77 ± 0.02 × 10^2^, *p* = 0.863; gephyrin contacts: 15.1 ± 0.7 × 10^2^, *p* = 0.340). At least four mice were analyzed for each genotype described. Scale bar = 10 μm. *From herein, box plots contain lower and upper limits (“whiskers”), 1^*st*^ *and 3*^*rd*^ *quartile (edges of the box), median (white bar), mean (white dot), and outliers (black dots) of the dataset.*

We next examined the effects of the loss of BDNF on the localization of GABAergic synaptic proteins in the internal granular layer of the cerebellum. In P14 conventional *Bdnf*^*−/−*^ mice, we observed an approximately 46% reduction in the localization of gephyrin (a postsynaptic marker for GABAergic synapses), but no impairment of the localization of GAD65 or GAD67 (presynaptic markers for GABAergic synapses) within the developing IGL (Fig. 3A–F; data not shown). The decrease in gephyrin localization results in an approximately 3.5 fold decrease in apposition of GAD67 and gephyrin at inhibitory synapses in the IGL (Fig. 3A–F; data not shown). In prior work, we demonstrated that absence of TrkB does not affect total expression of gephyrin within the cerebellum^9^. Consequently, the absence of BDNF, similar to that of TrkB, likely controls gephyrin localization at synapses, but not its total expression level. At this developmental stage (P14), the localization of GAD67 was also not detectably reduced in mice lacking *TrkB* in the cerebellum^9^. As the conventional *Bdnf*^*−/−*^ mice die around 2 weeks after birth, we have not examined the cerebella of older mice. We have, however, deleted *Bdnf* more specifically within cerebellar precursors, using *Wnt1*::Cre^35^ and were surprised to observe that these mice had normal localization of GAD67 and gephyrin at inhibitory synapses in the IGL (Fig. 3G–L). The apposition of GAD67 with gephyrin (Fig. 3M–R) also remained comparable in mice lacking BDNF compared to controls. Similar results were obtained following deletion of *Bdnf* specifically in granule cells using *mα6*::Cre (data not shown). These observations suggest that within the IGL, there is either compensation for BDNF due to presence of other neurotrophins with the ability to activate TrkB (e.g. NT3, NT4) or that BDNF is supplied by cells located outside the cerebellum.

### Mossy fiber-derived BDNF regulates localization of GABAergic synaptic proteins in the internal granular layer

**Figure 4:**
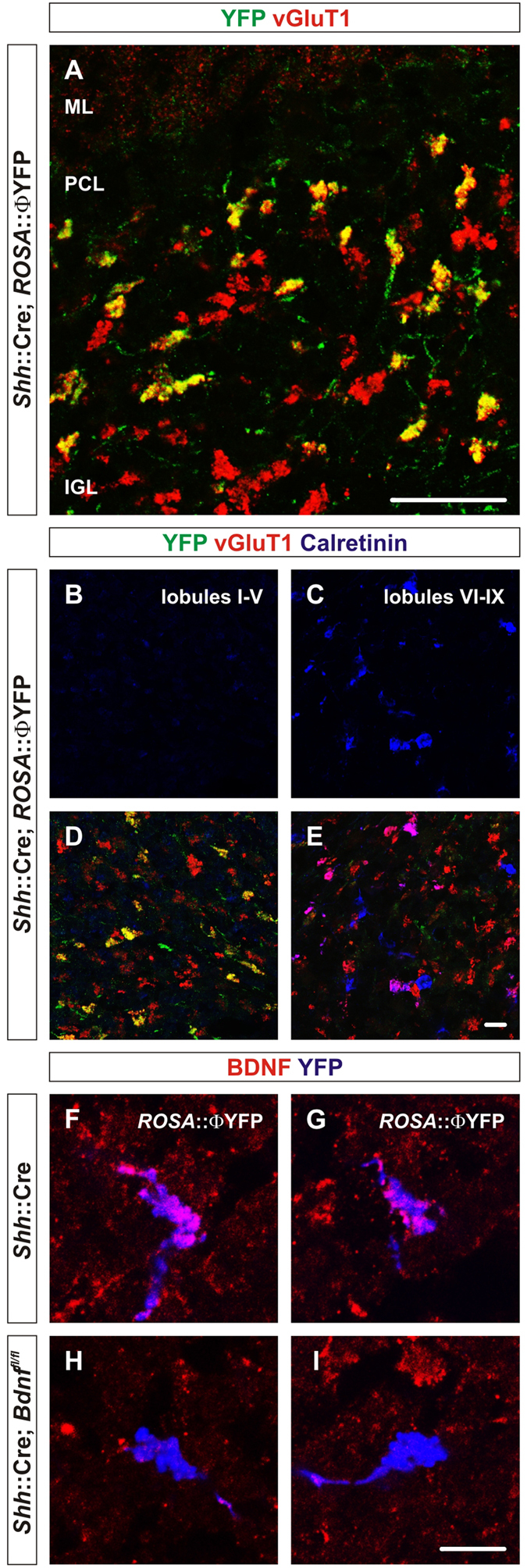
*Shh*::Cre mice recombine in a subset of mossy fibers derived from precerebellar nuclei and/or spinal cord neurons. (**A**) At P21, *Shh*::Cre mice selectively promote recombination in a subset of neurons that give rise to mossy fibers as assessed by YFP expression using the *ROSA*::ΦYFP reporter allele. Φ indicates a loxP-STOP-loxP cassette. Colocalization of vGluT1 expression with YFP^+^ mossy fibers (green) in P60 *Shh*::Cre; *ROSA*:: ΦYFP mice indicates that recombination occurs in a subset of mossy fibers (yellow). (**B,C**) The expression of Calretinin (blue) is mostly absent in lobule I-lobule V (**B**) and primarily found in lobule VI-lobule X (**C**). (**D,E**) The expression of YFP (green), which colocalizes with vGluT1, is found mostly in lobule I-lobule V (**D**), but not in lobule VI-lobule IX (**E**) in P60 *Shh*::Cre; *ROSA*::ΦYFP mice. Scale bar = 25 μm (**A**), 10 μm (**B–E**). (**F–I**) *Shh*::Cre mice effectively mediate deletion of BDNF in *Shh*::Cre; *ROSA*::ΦYFP; *Bdnf*^*fl/fl*^ mice. BDNF (red) is found in mossy fibers (blue) of *Shh*::Cre; *ROSA*::ΦYFP mice (**F,G**), but is absent in mossy fibers (blue) of *Shh*::Cre; *ROSA*::ΦYFP; *Bdnf*^*fl/fl*^ mice (**H,I**). Three mice of each genotype were analyzed. Scale bar = 50 μm (**A**), 10 μm (**B–I**). Φ indicates a loxP-STOP-loxP cassette.

In addition to granule cells, BDNF is expressed by neurons located outside the cerebellum that give rise to mossy fibers^36^. To explore the possibility that mossy fibers are the source of BDNF that controls gephyrin localization at Golgi cell-granule cell GABAergic synapses, we used mice that express Cre from the endogenous *Shh* locus^37^, which mediates recombination in neurons that give rise to a subset of cerebellar mossy fiber endings (Fig. 4A). YFP expression from the *ROSA*::YFP reporter was used to identify mossy fibers from neurons that have been recombined. Only 26 ± 3% of the mossy fibers in the IGL were recombined by *Shh*::Cre mice (Fig. 4A; data not shown). Mossy fibers terminals were recombined in lobules I-V, but not in lobules VI-IX (Fig. 4B–E). As expected, immunohistochemical analysis using anti-BDNF antibody showed that *Shh*::Cre efficiently deletes the *Bdnf* as well as the ROSA loci in mossy fibers (Fig. 4F–I).

Results showed that deletion of *Bdnf* from YFP^+^ mossy fibers did not reduce the localization of GAD67 at GABAergic synapses, but did result in reduced synaptic localization of gephyrin in P21 *Shh*::Cre; *Bdnf*^*fl/fl*^; *ROSA*::YFP mice (Fig. 4A–F). The mean area ratio of GAD67:vGluT1 expression was comparable between *Shh*::Cre; *Bdnf*^*fl/fl*^; *ROSA*::YFP mice and controls (Fig. 4C), but the mean area ratio of gephyrin:vGluT1 was reduced by ~54% in *Shh*::Cre; *Bdnf*^*fl/fl*^; *ROSA*::YFP mice compared to control (Fig. 4F). The requirement for BDNF is spatially restricted, as gephyrin localization is normal around mossy fibers originating from neurons that have not been recombined (Fig. 4G–L).

**Figure 5:**
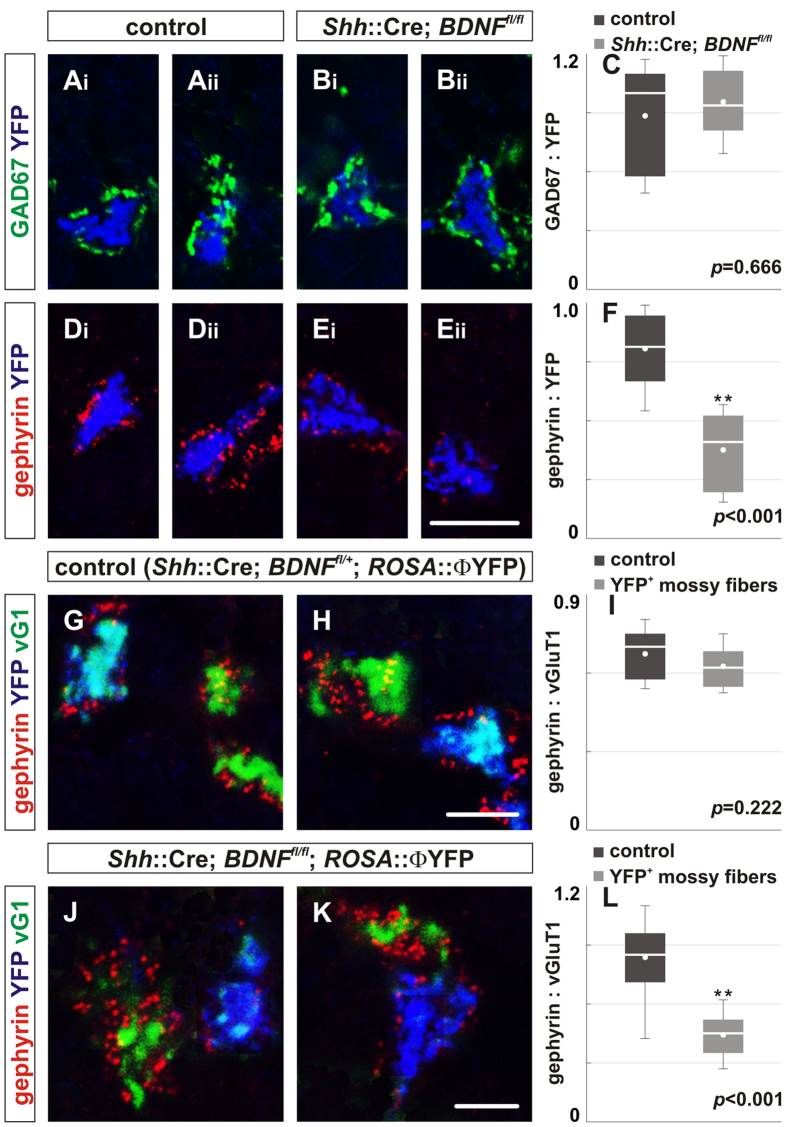
Postsynaptic differentiation at GABAergic synapses depends on mossy fiber-derived BDNF signaling. To explore the possibility that BDNF comes from an extracerebellar source, we deleted BDNF from a subset of mossy fibers and analyzed GABAergic synaptic proteins surrounding mossy fiber endings in the IGL. (**A–F**) YFP expression marks mossy fiber terminals from recombined neurons. Loss of BDNF in YFP^+^ mossy fiber endings did not influence GAD67 (green) localization in the IGL of P21 *Shh*::Cre; *Bdnf*^*fl/fl*^; *ROSA*::ΦYFP mice (Bi, Bii) compared to control (Ai, Aii). However, loss of BDNF in YFP^+^ mossy fiber endings resulted in reduced gephyrin (red) localization in the IGL of P21 *Shh*::Cre; *Bdnf*^*fl/fl*^; *ROSA*::YFP mice (Ei, Eii) compared to control (Di, Dii). (**C,F**) Quantification of the area ratio of GAD67:YFP (**C**) and the area ratio of gephyrin:YFP expression (**F**) in control (GAD67: 0.89 ± 0.09; gephyrin: 0.77 ± 0.05) and *Shh*::Cre; *Bdnf*^*fl/fl*^; *ROSA*::ΦYFP mice (GAD67: 0.97 ± 0.06, *p* = 0.666; gephyrin: 0.36 ± 0.05, *p* < 0.001). (**G–H**) The localization of gephyrin (red) surrounding vGluT1 between YFP^+^ mossy fibers (teal) and unrecombined YFP^-^ mossy fibers (green) is comparable in control mice. (**I**) Quantification of the area ratio of gephyrin:vGluT1 expression in P21 *Shh*::Cre; *Bdnf*^*fll+*^; *ROSA*::ΦYFP mice (YFP^-^ mossy fiber = 0.67 ± 0.01; YFP^+^ mossy fiber = 0.62 ± 0.01, *p* = 0.222). (**J,K**) However, the localization of gephyrin surrounding vGluT1 is reduced in recombined YFP^+^ mossy fibers (blue/teal) compared to unrecombined YFP^-^ mossy fibers (green) in mice lacking BDNF in a subset of mossy fibers. (**L**) Quantification of the area ratio of gephyrin:vGluT1 expression in P21 *Shh*::Cre; *Bdnf*^*fllfl*^; *ROSA*::ΦYFP mice (YFP^-^ mossy fiber = 0.84 ± 0.02; YFP^+^ mossy fiber = 0.44 ± 0.01, *p* < 0.001). More than four mice were analyzed for each genotype. Scale bar = 10 μm.

Interestingly, the expression of the vesicular glutamate transporter vGluT1 was also reduced within mossy fibers lacking BDNF (Fig. 5J–K) compared to control (Fig. 5G–H). We did not pursue this observation further to determine whether it is a cell-autonomous effect or whether it reflects alterations of innervations of the cell soma and dendrites of these neurons in the pons and hindbrain.

**Figure 6:**
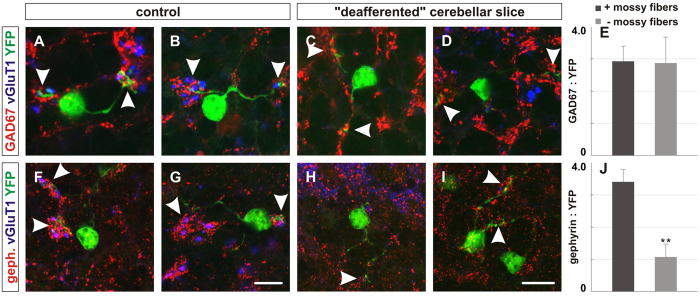
Loss of mossy fiber endings in cultured cerebellar slices perturbs postsynaptic differentiation. To assess whether the presence of mossy fibers or factors derived from mossy fibers regulate GABAergic synaptic differentiation, we generated cerebellar slices from P14 mice and cultured the slices *in vitro* for one week. (**A–D,F–I**) Cerebellar slices cultured *in vitro* for one week contain fewer vGluT1+ mossy fibers (blue) in the internal granular layer (C, D, H, I, white arrows) compared to age-matched control slices (A, B, F, G, white arrows). The localization of GAD67 (red) remains normal surrounding granule cell dendrites in “deafferented” cerebellar slices (**C,D**) compared to control (**A,B**). However, the localization of gephyrin (red) is significantly reduced in “deafferented” cerebellar slices (H, I) compared to control (**F,G**). (**E,J**) Quantification of the area ratios of GAD67:YFP and gephyrin:YFP expression in “deafferented” cerebellar slices (GAD67: 2.88 ± 0.79; gephyrin: 1.07 ± 0.37) compared to control slices (GAD67: 2.95 ± 0.48, *p* = 0.557; gephyrin: 3.4 ± 0.39, *p* < 0.001). At least four slices from three mice were analyzed for each condition. Scale bar = 20 μm.

To explore the impact of mossy fibers on GABAergic synapse formation in the IGL, we examined cerebellar slices cultured from P14-21 *in vitro* and compared these to P21 slices acutely prepared from 5 mice. Not surprisingly, the deafferented cerebellar slices cultured for 7 days *in vitro* (DIV) exhibited significantly reduced vGluT1^+^ expression compared to their age-matched controls (Fig. 6A–D,F–I). The localization of GAD67 surrounding granule cell dendrites was comparable between the cultured and control slices, but the localization of gephyrin was reduced by ~69% in cultured slices compared to the P21 controls (Fig. 6A–J). Together, these findings indicate that the presence of mossy fibers is required to direct localization of the postsynaptic scaffold protein, gephyrin, presumably because the mossy fibers are required for the delivery of BDNF.

### Addition of recombinant BDNF in primary cerebellar granule cells promotes clustering of key postsynaptic GABAergic proteins

**Figure 7:**
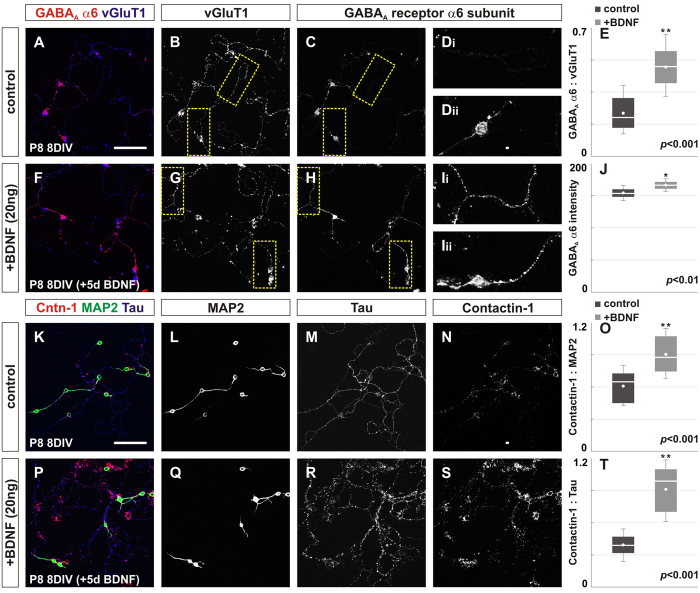
Exogenous BDNF increases the expression of the α6 subunit of GABA_A_ receptors and Contactin-1. (**A–J**) Addition of recombinant BDNF in primary cerebellar granule cell culture promotes the expression of the α6 subunit of GABA_A_ receptors. (**A,F**) Cerebellar granule cells are labeled by vGluT1 (blue) and the expression of GABA_A_R α6 subunit is shown in red. The expression of vGluT1 is not noticeably different after the addition of BDNF (**F,G**) compared to control (**A,B**). However, the expression of GABA_A_R α6 subunit is significantly increased after the treatment of BDNF for 5 days in culture (H, Ii, Iii) compared to control (C, Di, Dii). (**E**) Quantification of the area coverage of GABA_A_R α6 subunit over total vGluT1 in control (0.23 ± 0.13) and in culture after BDNF treatment (0.49 ± 0.17, *p* < 0.001). (**J**) Quantification of the intensity of GABA_A_R α6 subunit in control (155 ± 13) and in culture after BDNF treatment (168 ± 9.5, *p* < 0.01). (**K–T**) The expression of Contactin-1 on granule cell dendrites (MAP2, green, K, P) and axons (Tau, blue, K, P) was assessed following BDNF treatment. The expression of MAP2 is not noticeably different after the addition of BDNF (**Q**) compared to control (**L**), though the expression of Tau appears more punctate after the addition of BDNF (**R**) compared to control (**M**). However, the expression of Contactin-1 on the dendrites and axons of granule cells is significantly increased following BDNF treatment (**S**) compared to control (**N**). (**O**) Quantification of the area coverage of Contactin-1 over total MAP2 in control (0.61 ± 0.19) and in culture after BDNF treatment (0.91 ± 0.23, *p* < 0.001). (**T**) Quantification of the area coverage of Contactin-1 over total Tau in control (0.39 ± 0.15) and in culture after BDNF treatment (0.92 ± 0.28, *p* < 0.001). Scale bar = 50 μm.

To explore potential effector proteins that could mediate the activities of BDNF in postsynaptic differentiation, we assessed the consequences of adding recombinant human BDNF to cultured cerebellar granule cells. We previously showed that the localization of a cell adhesion molecule, Contactin-1, in the cerebellum is dependent on TrkB^9^ and others have shown that BDNF controls the translocation of Contactin-1 to the cell surface of hippocampal neurons^38^. We first examined the expression of a key postsynaptic GABAergic protein, the α6 subunit of GABA_A_ receptors in cerebellar granule cells obtained from P8 pups cultured for 8 days *in vitro*. We treated the cultured neurons with 20 ng of recombinant BDNF for 5 days starting at 3 days *in vitro*. We observed a ~2.1-fold increase in the expression of GABA_A_R α6 subunit in granule cells treated with BDNF along with an 8% increase in intensity of the protein expression (Fig. 7A–J). We next examined the expression of Contactin-1 and observed a ~1.5-fold increase in Contactin-1 on MAP2^+^ portions of the granule cells and a ~2.4-fold increase in Contactin-1 on Tau^+^ portions of the granule cells in cultures treated with BDNF (Fig. 7K–T). Consistent with this finding, our previous work showed that loss of Contactin-1 results in a reduction in the localization postsynaptic GABAergic proteins in the internal granular layer^9^. Taken together, we provide evidence that BDNF promotes the localization of key GABAergic postsynaptic proteins and supports the importance of Contactin-1 in the assembly and maintenance of cerebellar GABAergic synapses[Fig f8].

## Discussion

**Figure 8 f8:**
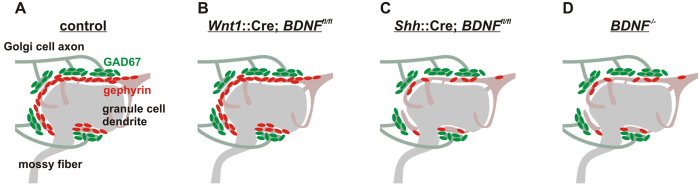
Summary of the effects of BDNF deletion on GABAergic synaptic protein localization. (**A**) The cerebellar glomerulus consists of mossy fiber terminals, granule cell dendrites and Golgi cell axons. GAD67 (green), a presynaptic GABAergic protein localized on Golgi cell axon endings, is adjacent to gephyrin (red), a postsynaptic GABAergic protein localized on granule cell dendrites. (**B**) Deletion of *BDNF* in the cerebellum did not result in any changes in the localization of GAD67 or gephyrin at the cerebellar glomerulus. (**C,D**) However, deletion of *BDNF* in mossy fibers (**C**) or conventional deletion of *BDNF* (**D**) resulted in reduced localization of gephyrin without any effects on the localization of GAD67.

Interference with BDNF expression results in defects in the development and function of GABAergic synapses^23^,^39^,^40^. Despite well documented roles of granule cell-expressed BDNF for migration of granule cell precursors and organization of synaptic vesicles within glutamatergic parallel fiber-GABAergic Purkinje cell dendrite synapses^15^,^41^, we found that deletion of *Bdnf* from granule cells or from all cells resident within the cerebellum did not impair the localization of GABAergic synaptic proteins within the internal granular layer (summarized in Figure 8). Interestingly, we found that loss of BDNF from a subset of mossy fibers resulted in reduced synaptic localization of gephyrin without influencing that of GAD65/67 (Figure 8). Since Golgi cells innervate multiple glomeruli^33^, GAD65/67 localization may be unaffected because the Golgi cells receive trophic BDNF support from adjacent glomeruli where mossy fibers express BDNF. Since *Bdnf* was only deleted from ~26% of mossy fiber terminals with the *Shh*::Cre used in the present work, it seems quite unlikely that individual Golgi cells were completely deprived of mossy fiber-derived BDNF. Thus, BDNF may control postsynaptic differentiation through local activation of TrkB, while BDNF controls presynaptic differentiation through more global mechanisms. Consistent with prior observations, our data indicate that granule cells are the major, probably the only cell type within the cerebellar cortex that express BDNF. Additionally, similar to a recent report^14^, we found that BDNF is preferentially localized within axons, but not dendrites of granule cells. Granule cell axon BDNF controls the apposition of pre- and postsynaptic specializations formed by GABAergic stellate and basket cells with Purkinje cell dendrites in the molecular layer. This observation is consistent with our prior work that demonstrated an important role for TrkB in promoting GABAergic synapse formation in the molecular layer^12^.

Several mechanisms could explain the specificity of granule cell- versus mossy fiber-derived BDNF in organizing cerebellar circuits. BDNF localization is controlled by mRNA transport, protein incorporation into dense core vesicles, and dense core vesicle transport and localization^14^,^18^,^20^. Its secretion is regulated by sortilin, CAPS1/2 and other proteins that control its incorporation into dense core vesicles, proteases that regulate proBDNF processing, and proteins, such as the syntaxins, CAPS2 and the synaptotagmins that regulate dense core vesicle exocytosis^5^,^14^,^19^,^20^,^24^,^40^. Processing of proBDNF to matureBDNF is essential for TrkB activation, because proBDNF activates only p75NTR, not TrkB, resulting in distinct consequences such as axon pruning and LTD^42^,^43^. Of special interest, recent work suggests that CAPS2 is preferentially localized to axons and is essential for axonal localization and normal exocytosis of BDNF^14^. Deletion of exon 3 within the p150Glued interaction domain of CAPS2 prevents localization of CAPS2 and BDNF to axons and results in a deficit in BDNF secretion^14^. CAPS2 transcripts lacking exon 3 have been identified in a few autistic patients and mice expressing CAPS2 lacking this exon exhibit autistic phenotypes, raising the interesting possibility that the specificity with which BDNF organizes CNS circuits described in this paper is important for organizing neural circuits elsewhere within the CNS and for expression of human cognitive and social behaviors that are abnormal in autism^14^,^44^.

BDNF controls the postsynaptic differentiation of excitatory synapses in the CNS by promoting dendritic spine formation in various brain regions such as the cortex, hippocampus and striatum^4^,^8^,^45^. Since BDNF is secreted as a consequence of neuronal activity, BDNF secretion is an important mechanism for controlling morphogenesis of dendritic spines in response to synaptic activity^46^. Data in the present work show that BDNF secreted by excitatory neurons additionally controls inhibitory synapse formation through regulation of gephyrin localization. While we did not examine the impact of BDNF deficiency on the total expression level of gephyrin, in prior work we demonstrated that absence of its receptor, TrkB, did not result in reduced expression of gephyrin within the cerebellum^9^. Thus we believe that the major effect of BDNF is to control gephyrin localization, not expression level in the cerebellum. In prior work, we have shown that activation of the BDNF receptor TrkB is important for both pre- and postsynaptic differentiation and maintenance of GABAergic synapses in the cerebellum^9^,^12^. In the IGL, the TrkB ligand, BDNF, secreted by mossy fibers regulates the clustering of gephyrin on granule cell dendrites; and in the molecular layer, BDNF secreted from granule cells regulates gephyrin clustering on Purkinje cell dendrites. In each instance, BDNF secreted by one neuron acts to promote GABAergic synaptic contact formation between two separate populations of cells. One potential molecular mechanism by which BDNF promotes gephyrin clustering could be through interactions of BDNF and Wnt signaling. Recent *in vitro* studies have shown that BDNF promotes the expression of Wnt2 which in turn promotes cortical dendritic growth and spine formation^47^. Both cerebellar granule cells and Purkinje cells express *Wnt* family members which could potentially work synergistically with BDNF to control GABAergic synapse formation^24^.

Postsynaptic scaffolding proteins such as gephyrin and PSD-95 organize neurotransmitter receptors to regulate the synaptic stabilization and homeostasis^48^,^49^. Prior work has shown that BDNF regulates PSD-95 at excitatory synapses through several pathways activated through TrkB, PI3Kinase-Akt, phospholipase-Cγ and MAPK/ERK signaling^50^, which control PSD95 palmitoylation and phosphorylation^50^. In prior studies, we obtained evidence that the PLC-γ binding site on TrkB is required for recruitment of gephyrin to GABAergic postsynaptic sites along granule cell dendrites^9^, implicating calcium-dependent pathways as effectors of TrkB action. Consistent with this, more recent and definitive work implicates calcium-calmodulin-dependent protein kinase II in phosphorylation-dependent regulation of gephyrin clustering^51^. Gephyrin localization is also controlled by collybistin, a phosphatidylinositol-3 phosphate binding protein with Rho family GEF activity, which is activated by binding to Neuroligin-2 as well as by the monomeric Cdc42 homologue CT-10/RhoQ^52^. Since phosphatidylinositide signaling and Cdc42 are activated by BDNF^53^, it seems likely that BDNF-TrkB control inhibitory synapse formation and maintenance through pathways in addition to those downstream of PLC-γ and calcium^52^.

## Methods

### Mouse Strains

Mouse strains used: *Wnt1*::Cre^35^, *mα6*::Cre^9^, *Shh*::eGFP-Cre^37^, *Bdnf*^*−/−*^54, *Bdnf*^*fl/fl*^^55^, *ROSA*::lacZ^56^, *ROSA*::ΦYFP^57^, *Thy1*::YFP-H-line^58^, *Bdnf*::lacZ^32^. All animals were maintained in the Laboratory Animal Resource Center (LARC) Rodent Barrier Facility at UCSF and Agency for Science, Technology and Research (A*STAR) Biological Resource Centre (BRC). All animal procedures were approved by the Institutional Animal Care and Use Committee (IACUC) following National Institutes of Health and A*STAR BRC animal care guidelines. Both sex from wildtype and all genotypes were used for analysis.

### Immunohistochemical analysis

Immunohistochemistry was performed on 15–20 μm cryosections as described (Rico *et al.* 2002) using fluorophore-conjugated secondary antibodies (1:500 to 1:1000; Jackson Immunoresearch and Molecular Probes, Invitrogen). Tissues from control and experimental conditions of mice with either sex were placed on the same slide to ensure all tissues were processed the similarly. Primary antibodies used in this study: monoclonal mouse anti-mature BDNF (Mab#9)^31^, guinea pig anti-vGluT1 (1:20000; Chemicon); mouse anti-gephyrin (1:8000; Synaptic Systems/SySy); rabbit anti-gephyrin (1:1000; SySy); rabbit anti-vGluT1 (1:10000) and rabbit anti-GAD65 (1:8000) (gifts from T. Jessell; see ref. 39); mouse anti-GAD67 (1:10000; Chemicon); sheep anti-GFP (1:500; Biogenesis); rabbit anti-GFP (1:1500; Molecular Probes); rabbit anti-Calretinin (1:5000; Swant); goat anti-β-galactosidase (1:1000; AbD Serotec). We found that the staining pattern for wildtype and experimental animals for all antibodies used in our studies did not differ between male or female.

For tissue staining with anti-mature BDNF (Mab#9) (Figs 1 and 4), brain tissues were immersed in an antigen retrieval solution (10 mM sodium citrate buffer, pH 6.0) at 4°C overnight. The tissues were then immersed in boiling antigen retrieval solution (200–500 ml) with stirring using a hot plate for 3–5 min. The tissues were then placed in cold 30% sucrose in PBS and treated with standard immunohistochemistry procedure.

### Quantification of synaptic protein expression and synaptic protein contacts

To determine the area and average pixel intensity of the expression of synaptic proteins surrounding individual mossy fiber endings within the IGL in lobules I-IV of the cerebellum^12^, images of a fixed area in the IGL from different mice were collected on a Zeiss LSM 5 Pascal confocal microscope (63x and 100x objectives, n.a. 1.40, Plan-Apochromat), keeping individual pixel intensities in the linear range for control images. The mean area (sum of pixels above threshold) and fluorescent pixel intensity of the protein of interest surrounding an individual glomerulus within each analyzed region were determined on cryosections at sub-saturating antibody concentrations and calculated using the histogram and color histogram function in NIH ImageJ (version 1.38x; NIH, USA).

For analysis shown in Figs 3 and 7, the sum of pixels above threshold was measured in an area of 21374 μm^2^ (63x objective) for each protein of interest and represented as an absolute number as previously described (Rico *et al.* 2002). For all other analysis, the sum of pixels above threshold was normalized to the sum of pixels of vGluT1 expression in an area of 8600 μm^2^ (100x objective) for each protein of interest and represented as an area ratio. The threshold was determined by measuring the average pixel intensity of an area in the cerebellar cortex that does not express the protein of interest. For analysis of the colocalized expression of BDNF (Fig. 1), colocalization of BDNF on individual cell-type is measured by using the *RG2B Colocalization* macro in ImageJ and BDNF expression not localized on cell type-specific markers was omitted using the same macro (http://rsbweb.nih.gov/ij/plugins/rg2bcolocalization.html).

For analysis shown in Figs 3 and 7, the number of contacts between synaptic proteins were calculated using a macro previously described^59^. This macro analyzes aspects of synapses by identifying apposing pre- and postsynaptic puncta of sufficient size and signal intensity, and objectively processes images with the same settings.

### Cerebellar slice culture

Organotypic cerebellar slices were obtained from P14 mice of either sex and cultured for 1 week based on previously described methods^60^. Brain dissection and slice preparation were carried out in low sodium Artificial Cerebrospinal Fluid (ACSF) containing 1 mM calcium chloride, 10 mM D-Glucose, 4 mM potassium chloride, 5 mM magnesium chloride, 26 mM sodium bicarbonate, 246 mM sucrose and phenol red solution (1:1000), ph 7.3. The solution was sterilized by filtering through a 0.22 μm filter and stored at 4 °C. Cerebellar slices were cultured in 75% Minimum Essential Medium Eagle (MEM), 25% heat-inactivated horse serum, 25 mM HEPES, 1 mM glutamine, 5 mg/ml glucose, penicillin (100 U/ml) and streptomycin (100 U/ml). After vibratome sectioning (Leica), the cerebellar slices were transferred to 6-well plates containing 30 mM culture plate inserts with 0.4 μm pores. 1 ml of culture media was added per well which was pre-conditioned by incubation for at least 2 hours in the cell culture incubator at 37 °C and 5% CO_2_.

### Mixed cerebellar neuronal culture

Cerebella were dissected from P5 mice of either sex in cold CMF-PBS, then mechanically minced into smaller pieces. Intact tissue was collected in 15 ml polypropylene tube containing 2.5 ml cold CMF-PBS. 250 μl of 10X trypsin and 10X DNAse were added to digest the cerebella for 10–15 min. at 37 °C. The cells were centrifuged at 800 x RCF at 4 °C for 5 minutes to remove the trypsin. Cells were resuspended in 2 ml CMF-PBS (with 200 μl 10X DNAse). The cells were triturated with P1000 tips and the total volume was brought up to 5 ml. Cells were filtered (70 μm nylon cell strainer) into 50 ml falcon tube and centrifuged at 900 x RCF at 4 °C for 5 minutes to remove debris. Cells were then resuspended in 5 ml medium (+serum). Cells were plated on glass coverslips pretreated with poly-D-lysine/poly-L-lysine at a density of 1 × 10^6^ cells/ml. Medium was changed to serum-free medium next day. Half the medium was changed every 2–3 days. Recombinant human BDNF was generously provided by Amgen (Thousand Oaks, CA).

### Analysis and quantification of immunogold labeling with electron microscopy

P60 wildtype mice of either sex were perfused and postfixed overnight using 4% paraformaldehyde. Cerebellar tissues were processed as previously described^9^,^12^. We analyzed individual glomeruli from 20 electron micrographs (magnification of 11,500X, single 2 μm sections). Only simple glomeruli were analyzed since large complex glomeruli containing more than one mossy fiber ending extended outside of the micrographs. Individual components of each glomerulus were identified by criteria described previously^9^ using those published by others^33^,^34^.

## Additional Information

**How to cite this article**: Chen, A. I. *et al.* Glutamatergic axon-derived BDNF controls GABAergic synaptic differentiation in the cerebellum. *Sci. Rep.* **6**, 20201; doi: 10.1038/srep20201 (2016).
